# CAEM-GBDT: a cancer subtype identifying method using multi-omics data and convolutional autoencoder network

**DOI:** 10.3389/fbinf.2024.1403826

**Published:** 2024-07-15

**Authors:** Jiquan Shen, Xuanhui Guo, Hanwen Bai, Junwei Luo

**Affiliations:** School of Software, Henan Polytechnic University, Jiaozuo, China

**Keywords:** cancer subtype, cancer subtype identification, convolutional autoencode, convolutional block attention module, multi-omics

## Abstract

The identification of cancer subtypes plays a very important role in the field of medicine. Accurate identification of cancer subtypes is helpful for both cancer treatment and prognosis Currently, most methods for cancer subtype identification are based on single-omics data, such as gene expression data. However, multi-omics data can show various characteristics about cancer, which also can improve the accuracy of cancer subtype identification. Therefore, how to extract features from multi-omics data for cancer subtype identification is the main challenge currently faced by researchers. In this paper, we propose a cancer subtype identification method named CAEM-GBDT, which takes gene expression data, miRNA expression data, and DNA methylation data as input, and adopts convolutional autoencoder network to identify cancer subtypes. Through a convolutional encoder layer, the method performs feature extraction on the input data. Within the convolutional encoder layer, a convolutional self-attention module is embedded to recognize higher-level representations of the multi-omics data. The extracted high-level representations from the convolutional encoder are then concatenated with the input to the decoder. The GBDT (Gradient Boosting Decision Tree) is utilized for cancer subtype identification. In the experiments, we compare CAEM-GBDT with existing cancer subtype identifying methods. Experimental results demonstrate that the proposed CAEM-GBDT outperforms other methods. The source code is available from GitHub at https://github.com/gxh-1/CAEM-GBDT.git.

## 1 Introduction

As a branch of the disease, cancer is a complex genomic disease that can occur in various parts of the human body and has a great impact on human life. Therefore, cancer treatment and prognosis are very important, but accurate identification of cancer subtypes is an important prerequisite when cancer treatment and prognosis are carried out (Guo et al., 2023). Patients with the same subtype of cancer may behave differently (Alexander et al., 2022), but they share common characteristics that can be used to identify them. Accurate identification of cancer subtypes is conducive to exploring the pathogenesis of cancer, plays a decisive role in treatment and prognosis (Zhang et al., 2022), greatly increases the cure rate of patients, and promotes the further development of research on cancer subtype identification (Wang et al., 2020).

The mainstream bioinformatics data used by researchers is multiple types of omics data based on tumors. These data express cancer from different perspectives. Multi-omics data include genome, transcriptome, epigenome, proteome, exposome, and microbiome (Ao et al., 2022). Multi-omics data analysis can reveal different aspects of the same sample and provide additional beneficial information for research. Therefore, compared with a single type of gene expression data, methods based on multi-omics data can more accurately identify cancer subtypes.

Gene expression data in bioinformatics refers to the data that describe the expression levels of genes under specific conditions or in specific cell types. Gene expression is the process by which genetic information is transcribed into RNA and translated into proteins. miRNA molecules participate in the regulation of gene expression mainly by binding to target mRNA, leading to their degradation or translation inhibition, thereby regulating gene expression. DNA methylation data provides information about the methylation status in the genome. These data can reveal which genes or gene regions are methylated under specific conditions or in different types of cells. DNA methylation data plays a crucial role in studying gene regulation. The relationship between these three types of omics data is inseparable. They influence each other, and the relationship between them can be extracted through models to achieve the identification of cancer subtypes. Therefore, ultimately, these three types of omics data are adopted as inputs to the model.

The main challenge currently is how to extract the information related with cancer subtypes from multi-omics data. Meanwhile, the dataset about cancer patients usually includes a small number of samples, and the samples have very high dimensions (Brigham et al., 2012; Hammerman et al., 2012; Muzny et al., 2012).

Most of the existing methods are based on unsupervised learning clustering or supervised learning classification, using multi-omics data to achieve cancer subtype identification. It can be divided into early integration, intermediate integration and late integration (Lipkova et al., 2022) according to the different focus of the identification method. Pre-integration simply links mult-omics data into one matrix, and then processes the unified matrix. These methods commonly exacerbated the problem of dimensionality explosion, making imbalanced phenomenon of sample size and dimensionality even more serious. The representative methods include K-means, Spectral clusting and LRAcluster (low-rank-approximation-based multi-omics data clustering) (Wu et al., 2015). The characteristic of this type of method is that it is easy to ignore the local characteristics of some omics data (Zhao et al., 2023).

The method of post-integration is different from the method of pre-integration. Post-integration processes each omics data separately and integrates the clustering results to obtain the final clustering solution. Post-integration effectively avoids the mistakes of pre-integration. Clustering is performed on each omics data to ensure the influence of weak signals. Such as moBRCA-net (Choi and Chae, 2023) and Subtype-WESLR (Song et al., 2022), but post-integration also has its own shortcomings. When multiple omics data have different contributions to the clustering results, it will have a great impact on the performance of these method. So both pre-integration and post-integration have a common characteristic, they are unable to express interactions between different omics data. Therefore, mid-integration has gradually become a favored object among researchers (Kang et al., 2022).

Mid-integration achieves data merging and dimensionality reduction by establishing an unified model. Not simply linked data or linked feature matrices. iCluster Bayes (Mo et al., 2018) and Cascade Deep Forest (El-Nabawy et al., 2021) present statistical ensemble methods to solve ensemble challenges. These methods model the distribution of each data type and then maximize the likelihood of multi-omics data based on joint latent variables. However, due to the complexity of multi-omics data, traditional statistical or mathematical models still face huge challenges in accurately modeling high-dimensional multi-omics data. The earliest method SNF (Wang et al., 2014), ERDCN (Lei et al., 2022) process the multi-omics data by constructing a sample similarity network about the co-expression patterns of cancer genes. However, these methods are susceptible to data noise and feature heterogeneity.

In recent years, deep learning has become more and more widely used in the field of medical care and has become a popular method favored by researchers (Dai et al., 2021). Many of these models have achieved good results in the field of cancer subtype identification, such as HI-DFN Forest (Xu et al., 2019), Subtype-GAN (Yang et al., 2021) and SADLN (Sun et al., 2023). The HI-DFN Forest employs a stacked autoencoder to learn advanced representations from each omics data, and then integrates all the learned representations into an autoencoder layer to learn complex representations. The DFN Forest model classifies (Zhou and Feng, 2019) patients into different cancer subtypes. Subtype-GAN utilizes a generative adversarial network to extract features from omics data through relatively independent layers and simultaneously input the extracted information into a shared layer for data integration. Consensus GMM clustering is used to predict cancer subtype outcomes. SADLN combines the encoder, self-attention, decoder, and discriminator into a unified framework, leveraging the integrated representations learned from the network, and utilizes Gaussian mixture models to identify cancer subtypes.

In recent years, convolutional autoencoders have shown good performance in reducing the dimensionality of high-dimensional data (Naderan and Zaychenko, 2020). applied convolutional autoencoders to the field of cancer subtype identifying and conducted comparative experiments with regular autoencoders on image datasets. The experimental results indicate that convolutional autoencoders achieve better dimensionality reduction compared to traditional autoencoders.

In this paper, we propose a method called CAEM-GDBT, which combines a convolutional autoencoder, a convolutional self-attention module, and a GBDT classifier to accomplish the classification of cancer subtypes. CAEM-GBDT is dedicated to establishing an integrated framework that includes an encoder, decoder, attention module, and identification module, enabling joint training and optimization. Compared to existing methods, it requires fewer computational resources and offers higher accuracy. The limitation of early integration is that the feature extraction module and the subsequent clustering module are relatively independent. In contrast, in CAEM-GBDT, the feature extraction module is tightly connected with the identification module. The drawback of late integration is that when extracting features, weights are introduced to process multi-omics data, which can easily overlook the interactions between different types of omics data. CAEM-GBDT employs a convolutional attention module mechanism, processing features from both channel and spatial perspectives, fully leveraging the relationships between different omics data. It treats multi-omics data as a whole for feature extraction. In summary, CAEM-GBDT can overcome the limitations of both early and late integration.

## 2 Methods

**FIGURE 1:**
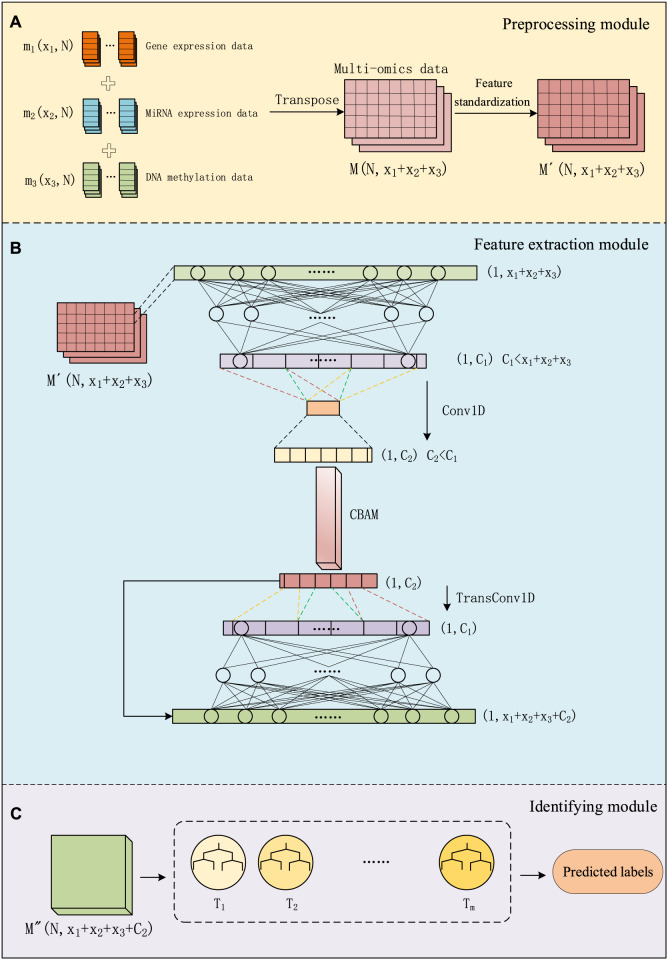
The process of CAEM-GBDT, **(A)** represents the data Preprocessing module, **(B)** represents the Feature extraction module, **(C)** represents the Identifying module.

We divided the entire method into three modules: data preprocessing module, feature extraction module, and identifying module. As shown in Figure 1, in the data preprocessing module, we preprocess multi-omics data and use it as input for the next module. In the feature extraction module, we use a combination of a convolutional autoencoder and a convolutional attention module for extracting important features about cancer subtypes. In the convolutional autoencoder. We use one-dimensional convolutional layers to replace some of the fully connected layers in a regular encoder. By employing one-dimensional convolution, we extract low-dimensional key features from the data. This feature is then connected to the output of the decoder to form a matrix, and this combined input is fed into the classifier section. In the identifying module, a GBDT classifier is used for cancer subtype identifying. This entire process is referred to as CAEM-GBDT.

### 2.1 Preprocessing module

We use three types of omics data as inputs to the model, including the gene expression data matrix *m*_*1*_(*x*_*1*_ *, N*), miRNA expression data matrix *m*_*2*_(*x*_*2*_ *, N*) and DNA methylation data matrix *m*_*3*_(*x*_*3*_ *, N*), Each column represents a sample, with a total of *N* samples, and rows *x*_*1*_, *x*_*2*_ and *x*_*3*_ represent the dimensions of different data types. We transpose and concatenate these data matrices into a multi-omics data matrix *M*.

For multi-omics data with different dimensions, we concatenated the multi-omics feature matrices from the perspective of sample quantity. Although different omics data within the same dataset have different sample dimensions, they have the same number of samples. We concatenate the multi-omics data from the perspective of sample number and, when inputting to the model, designate different encoder networks for data with different dimensions. This approach enables the concatenation of multi-omics data.

As shown in Figure 1A, before training the model, the data matrix undergoes feature standardization aiming to map features to a distribution with a mean of 0 and a standard deviation of 1. The multi-omics matrix after standardization is denoted as $M^{\prime }$ . Specifically, we calculate the mean $u_{i}$ and standard deviation $\sigma_{i}$ for the i-th sample, the feature standardization Formula is defined as Formulas 1 and 2:

$$\sigma_{i}=\sqrt{\frac{{\left(X_{i1}-u_{i}\right)}^{2}+{\left(X_{i2}-u_{i}\right)}^{2}+\ldots {\left(X_{in}-u_{i}\right)}^{2}}{n_{i}}}$$ (1)

$$X_{ij}^{\prime }=\frac{X_{ij}-u_{i}}{\sigma_{i}}$$ (2)

the calculation is applied to each row, where $X_{ij}$ represents the j-th dimension of the i-th sample, and $X_{ij}^{\prime }$ represents the data after feature standardization. The final preprocessed matrix $M^{\prime }$ is composed of the elements $X_{ij}^{\prime }$ .

### 2.2 Feature extraction module

#### 2.2.1 Convolutional autoencoder

The autoencoder, operates by mapping input data through a multi-layer network. In this process, we first process the matrix by an encoding step and extract intermediate features. The features are reconstructed with the same size as the input. The process involves compressing the data (encoding), followed by using this complex representation to reconstruct the data (decoding). Throughout the learning process, optimization of the loss function occurs to minimize it, aiming to faithfully reconstruct the input. Through the autoencoder, the system learns meaningful representations of the input data, which is valuable for tasks such as feature learning and data reconstruction.

As shown in Figure 1B, Given the input sample $X_{i}$ from matrix $M^{\prime }$ , the high-level representation extracted from the input layer through several fully connected layer functions. Such as the first fully connected layer function $g_{1}\left(X_{i}\right)$ , the corresponding high-level representation is denoted as $h_{i}$ , $g_{1}\left(X_{i}\right)$ can be expressed as Formula 3:

$$h_{i}=g_{1}\left(X_{i}\right)=\sigma \left(W\cdot X_{i}+b\right)$$ (3)

where ( W,b ) represents the parameters during the training process of the autoencoder. $X_{i}$ represents the input to the encoder. Dense layer can fully utilize information in the network during feature extraction, but it requires more time and resources for training. Therefore, the main feature extraction task is still handled by the convolutional layers.

Next, the high-level representation *h* from several fully connected layer is processed through several Conv1D layers. In the calculation process of convolution, the output data $\left(N,W_{2},H_{2},D_{2}\right)$ is determined by the input data $\left(N,W_{1},H_{1},D_{1}\right)$ , *N* represents the number of samples, and $D_{2}=k$ , Formulas 4 and 5 are used to calculate *W*_*2*_ and *H*_*2*_ are as follows:

$$W_{2}=\frac{W_{1}-f+2p}{s}+1$$ (4)

$$H_{2}=\frac{H_{1}-f+2p}{s}+1$$ (5)

*W*_*1*_ represents the width of the input data, and *H*_*1*_ represents the height of the input data. The convolution kernel size f , the stride s , the number of convolution kernels k and the amount of zero-padding p . In the convolution layer, padding is set to “same” mode, which represents the output height and width as the input size divided by the stride, rounded up. The advantage of Conv1D in its ability to extract local features from input sequences while preserving the order information of the sequence. Compared to fully connected layers, Conv1D can reduce the number of parameters, decrease model complexity, and enhance the model’s generalization ability.

Finally, the input sample $X_{i}$ undergoes processing through several fully connected layers and Conv1D layers, transforming into ${X_{i}}^{\prime }$ . The dimension of ${X_{i}}^{\prime }$ is much lower than the dimension of the input sample. ${X_{i}}^{\prime }$ is further enhanced through the CBAM attention mechanism, resulting in ${X_{i}}^{\prime \prime }$ . ${X_{i}}^{\prime \prime }$ and ${X_{i}}^{\prime }$ have the same size. ${X_{i}}^{\prime \prime }$ is then fed into the decoder section for reconstruction.

In the decoder section, the low-dimensional feature ${X_{i}}^{\prime \prime }$ undergoes several transposed convolution layers and fully connected layers to restore its original size. A transposed convolutional layer comprises a conv1DTranspose layer and an upsampling layer. Formula 6 is used to calculate conv1DTranspose layer:

$$Outsize=stride\times \left(Insize-1\right)+kernel\_size-2*padding$$ (6)

where *Insize* represents the input height or width, *padding* represents the padding size. In transposed convolution, the purpose of padding is to remove the outermost layer. *Stride* represents the step size, *kernel_size* is the size of the convolutional kernel, and *Outsize* is the height or width output after transposed convolution. The upsampling layer, in contrast to the max-pooling layer, repeats each time step size times along the time axis, restoring the dimensions scaled by max-pooling. The data processed by the transposed convolutional layers is denoted as ${X_{i}}^{\prime \prime \prime }$ .

Following, the high-level representation ${X_{i}}^{\prime \prime \prime }$ is processed through the last few fully connected layers. Such as the first fully connected layer function $g_{2}\left(X_{i}^{\prime \prime \prime }\right)$ . $g_{2}\left(X_{i}^{\prime \prime \prime }\right)$ can be expressed as Formula 7:

$$X_{i}^{⁗}=g_{2}\left(X_{i}^{\prime \prime \prime }\right)=\sigma \left(W^{\prime }\cdot X_{i}^{\prime \prime \prime }+b^{\prime }\right)$$ (7)

where $W^{\prime },b^{\prime }$ have the same meaning as W,b in the encoding section, they are trainable parameters in the fully connected layers. Finally, the low-dimensional features ${X_{i}}^{\prime \prime }$ extracted by CBAM and the fully connected features $X_{i}⁗$ are concatenated as the output of the CAEM section. These outputs together form the matrix $M^{\prime \prime }$ .

We chose the GELU (Gaussian Error Linear Unit) activation function for both the convolutional layers and transposed convolutional layers. GELU can be seen as a combination of the relu and dropout concepts. For high-dimensional biological data, an abundance of features may impact feature learning. In such cases, if there is a desire to discard unimportant information, the GELU activation function is employed for non-linear transformation of features. When x is relatively large, y is more likely to be retained, and as x decreases, y is more likely to be set to 0. However, when x is less than 0, there is a certain probability that y is not set to 0. The mathematical formula for the approximate computation of GELU is Formula 8:

$$\text{GELU}\left(x\right)=0.5x\left(1+\tanh\left(\sqrt{2/\pi }\left(x+0.044715x^{3}\right)\right)\right)$$ (8)

The structure of the layered convolutional encoder involves constructing a CAE for each type of omics data. The hyperparameters of each CAE are optimized separately to make the reconstructed input of each CAE as similar as possible to its original input. Ultimately, the goal is to find a model that performs well on a combination of various omics data types. The cost function for each CAE is formulated as Formula (9):

$$J_{CAE}=\frac{1}{2N}{\left(x_{i}-x_{i}^{\prime }\right)}^{2}+\lambda {\left\|M^{\prime }\right\|}_{2}^{2}$$ (9)

Where the first term represents mean squared error, and the second term is L2 regularization, $x_{i}$ represents the original input data, $x_{i}^{\prime }$ represents the data reconstructed by the CAE, *N* represents the number of samples, λ is the parameter of the L2 regularization term, and $M^{\prime }$ is the input matrix. The training process for each CAE involves using the gradient descent algorithm to minimize the two components of the loss. The proposed model’s layered convolutional encoder structure minimizes the cost functions of three CAEs through gradient descent, thereby optimizing the parameters of the entire model.

#### 2.2.2 Convolutional attention

**FIGURE 2:**
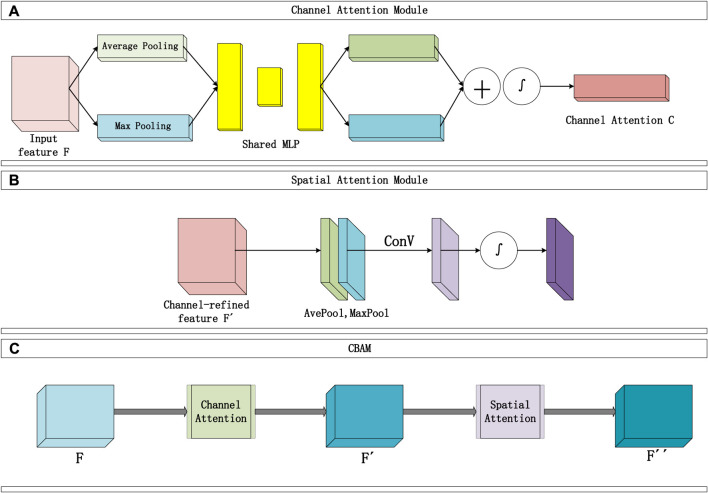
Convolutional attention module structure diagram, **(A)** represents the Channel Attention module, **(B)** represents the Spatial Attention module.

The convolutional block attention module consists of two sequential parts, as shown in Figure 2: the Channel Attention Module (Figure 2A) and the Spatial Attention Module (Figure 2B). The input data typically includes multiple channels. The Channel Attention Module’s main function is to assign individual weights to each channel, enhancing the influence of important channels and reducing the proportion of irrelevant channel information. Two types of pooling layers are used to pool the input feature F in terms of width and height. The pooling results are then processed using a shared fully connected layer (MLP). Afterward, the results are integrated through addition, and the channel attention map C is obtained through a mapping function ∫. Finally, the weights are multiplied to channel-wise weight C and applied to F.

The Channel Attention Module constructs two shared fully connected layers (MLP) in the attention module, namely, ‘shared_layer_one’ and ‘shared_layer_two’. The specific structure is as follows: ‘shared_layer_one’ is the first fully connected layer, with an output dimension equal to the number of channels divided by the ratio, and uses the relu activation function. ‘Shared_layer_two’ is the second fully connected layer, with an output dimension equal to the number of input feature channels, and has no activation function. These shared layers are used to process the results of average pooling and max pooling, respectively calculating the weights of each channel.

In order to obtain an effective channel attention map, the dual pooling computation process enhances the feature representation compressed by this module. The specific computation process is illustrated in Figure 2A, and the calculation formula can be expressed as Formula 10:

$$C\left(F\right)=\int \left(\text{MLP}\left(\text{AvgPool}\left(F\right)\right)+\text{MLP}\left(\text{MaxPool}\left(F\right)\right)\right)$$

$$=\int \left(W_{1}\left(W_{0}\left(F_{avg}\right)\right)+W_{1}\left(W_{0}\left(F_{\max}\right)\right)\right)$$ (10)

Where ∫ represents the Sigmoid activation function, W_1_、W_0_ are the weight matrices shared by the MLP, and C(F) represents the channel attention map (weights).

Moreover, the use of shared layers ensures that the same set of weights is applied to different pooling results, thereby introducing consistency in the computation. The two shared fully connected layers are applied sequentially to the results of average pooling and max pooling, meaning that these fully connected layers are shared in both pooling paths. In this way, the average pooling and max pooling results are processed into the same dimensions and transformed by the same network weights. This shared mechanism ensures consistency when the model calculates the attention weights of each channel, avoiding the introduction of different parameters in different pooling paths, thereby enhancing the model’s generalization ability.

The Spatial Attention Module focuses on which information in the input data is more crucial and can further complement features that were not previously noticed. Specifically, this module sequentially performs two types of pooling computations along the channel direction, then stacks them to form a numerical matrix. Subsequently, it undergoes a standard convolutional layer (with one channel) to concatenate, adjusting the previously disordered channels in the matrix. Following that, an attention map S is calculated using an activation function, and finally, S is multiplied with the input of this module. The Spatial Attention Module is illustrated in Figure 2B, and the detailed calculation Formula 11 is as follows:

$$S\left(F^{\prime }\right)=\int \left(f^{7*7}\left(\left[\text{AvgPool}\left(F^{\prime }\right);\text{MaxPool}\left(F^{\prime }\right)\right]\right)\right)$$ (11)

In the equation, ∫ represents the Sigmoid function, $f^{7*7}$ represents a convolutional kernel with a size of 7 × 7, $F^{\prime }$ represents the result of multiplying the input data *F* by the channel attention map C, and $S\left(F^{\prime }\right)$ is the spatial attention map.

Finally, the overall process of the Convolutional Attention Module is illustrated in Figure 2B and can be summarized as follows: The input F passes through the first channel module to obtain the channel attention map C, and the matrix $F^{\prime }$ is obtained through multiplication. Subsequently, it goes through the second module to generate the spatial attention map S, and the final feature matrix $F^{\prime \prime }$ is obtained through the same multiplication. The calculation process is shown in formulas (12) and (13):

$$F^{\prime }=C\left(F\right)⊗F$$ (12)

$$F^{\prime \prime }=S\left(F^{\prime }\right)⊗F^{\prime }$$ (13)

CBAM combines the spatial attention map $S\left(F^{\prime }\right)$ obtained from the above two sub-modules with $F^{\prime }$ and multiplies them to get the scaled new features. In the proposed CAEM in this chapter, the high-level representation of a single CAE or the integrated high-level representation of three CAEs will be processed by CBAM to obtain a new feature matrix.

### 2.3 Identifying module

This study employed the GBDT classifier for cancer subtype identifying. GBDT is one of the excellent algorithms in the Boosting series of ensemble learning. Boosting imparts a strong dependency relationship among individual learners of the same kind, integrating them sequentially. The Gradient Boosting Tree algorithm organizes weak classifier decision trees through the addition principle, connecting each weak classifier. It optimizes residuals using gradient descent. In GBDT, the next weak classifier is trained using the gradient of the loss from the previous weak classifier. In this way, each iteration develops in the direction of reducing the loss, seeking an optimal solution. GBDT is an additive model of decision trees (as shown in Figure 1C), and the general calculation process can be represented as Formula 14:

$$f_{M}\left(x\right)=\sum_{m=1}^{M}T\left(x;\theta_{m}\right)$$ (14)

$T\left(x;\theta_{m}\right)$ represents a decision tree, where $\theta_{m}$ denotes the detailed parameters of the tree, and M represents the total number of trees.

GBDT adopts the forward distribution algorithm. The first step is to initialize the boosting tree. $f_{0}\left(x\right)=0$ . Then, the model at the m-th step is calculated using Formula 15:

$$f_{m}\left(x\right)=f_{m-1}\left(x\right)+T\left(x;\theta_{m}\right)$$ (15)

In Formula 16, the parameters of the next tree are chosen to minimize the bias as much as possible:

$$\theta_{m}=\arg\min_{\theta_{m}}\sum_{i=1}^{N}L\left(y_{i},f_{m-1}\left(x_{i}\right)+T\left(x_{i};\theta_{m}\right)\right)$$ (16)

“L ()" refers to the loss function. The loss function for GBDT classification algorithm is different from regression algorithms. It calculates the training loss using the formula of log-likelihood loss function, as shown in Eq. 17:

$$logloss=-\frac{1}{N}\sum_{i=1}^{N}\left(y_{i}\log\left(p_{i}\right)+\left(1-y_{i}\right)\log\left(1-p_{i}\right)\right)$$ (17)

Where *N* is the number of samples, $y_{i}$ is the probability of the first i samples being true positive samples, and $p_{i}$ is the probability of predicting positive samples for the first i samples.

GBDT adopts a one-vs-all strategy to predict multi-class targets. M decision trees are trained as weak classifiers for each different category. Assuming there are K categories, after training, there will be a total of M*K trees. The training objective of GBDT is to optimize gradients to reduce bias, thereby improving the final classifier’s results. Each decision regression tree has a limited depth. Finally, GBDT weights and sums the weak classifiers obtained in each round of training to obtain the predicted labels for cancer subtypes.

To address the potential overfitting issue in GBDT, we adopted the following two methods to reduce the risk: Limiting the number of weak classifiers: Generally, increasing the number of weak classifiers enhances the model’s fit to the training data but also increases the risk of overfitting. In our study, we limited the number of weak classifiers to 300, which helps reduce the overfitting risk. Early stopping strategy: By monitoring the model’s performance on the validation set, we save the parameters and stop training when the model reaches its optimal performance. This effectively prevents the model from overfitting the training data.

To ensure the model’s generalization ability, we integrated parameters from two datasets, enabling GBDT to adapt to both the BRCA and GBM datasets, thereby improving the model’s generalization capability. Additionally, the weak classifiers in GBDT consider only a subset of features each time, which, while reducing the correlation between features, enhances the model’s generalization ability.

In CAEM, the feature matrix obtained through the Convolutional Autoencoder and Convolutional Attention modules is input into the GBDT module to obtain the final prediction results for cancer subtypes.

## 3 Experiment and analysis

### 3.1 Datasets

To demonstrate the effectiveness of the proposed method CAEM-GBDT, this study considered two different datasets. One dataset is from TCGA, including 104 samples of Invasive Breast Carcinoma (BRCA), the another dataset includes 213 samples of Glioblastoma Multiforme (GBM). We randomly divide each dataset into five equally sized parts, with four parts used as the training set and the remaining one part as the test set. However, in the BRCA dataset, due to the small number of samples in the test set, we changed the ratio of the training set to the test set to 3:1 when the input data was multi-omics data. We set 100 epochs for training the model. For each cancer type, we utilized gene expression data, miRNA expression data, DNA methylation data. Table 1 shows detailed information about the two datasets.

**TABLE 1:** Information of the datasets.

Cancer type	Gene expression	miRNA Expression	DNA methylation	Multi-omics	Patient
BRCA	17,814	354	23,094	41,262	104
GBM	12,042	534	1,305	13,881	213

### 3.2 The encoder network structure and parameter settings

In order to choose a CAE structure with better performance, this study trained CAEs with different numbers of hidden layers and hidden units for different data types. Tables 2, 3 shows the corresponding network structures and parameters for CAE.

**TABLE 2:** The structure and parameters of CAE (BRCA).

Architecture	CAE (BRCA)	
Encoder	Input (Gene ExpressionDNA Methylation)	Input (miRNA Expression)
	512 (Dense)	128 (Dense)
	16 + 2(Conv1D + Maxpool)	4 + 4(Conv1D + Maxpool)
	4 + 4(Conv1D + Maxpool)	
Decoder	4 + 4 (TransConV + UpSampl)	4 + 4 (TransConV +UpSampl)
	16 + 2 (TransConV + UpSampl)	
	512 (Dense)	
	Output	Output

**TABLE 3:** The structure and parameters of CAE (GBM).

Architectures	CAE (GBM)
Encoder	Input (Gene Expression/DNA Methylation/miRNA Expression)
	128 (Dense)
	4 + 4(Conv1D + Maxpool)
Decoder	4 + 4 (TransConV + UpSampl)
	1 (TransConV)
	Output

Where the convolutional layers have a kernel size of 3, padding mode is set to ‘same’, and the stride is 2. The activation function for the convolutional layers is chosen as the Gelu function.

In the GBM dataset, we have adopted the same encoder structure for three different types of omics data. The main reason for this is that the dimensionalities of multiple omics data in the GBM dataset are not significantly different from those in the BRCA dataset. Therefore, in the BRCA dataset, we set the same encoder network for gene expression data and DNA methylation data, which have larger dimensions, while a different encoder network is set for miRNA data. This approach ensures that the convolutional autoencoder is better suited to each omics data type, leading to more efficient feature extraction.

In addition to CAE, for the GBDT module, this study employs five-fold cross-validation to calculate the minimum mean squared error for evaluating the performance of different parameter settings in GBDT. Table 4 presents the specific parameter settings for the GBDT module.

**TABLE 4:** The parameters of GBDT.

Parameter	Setting values
Learning_rate	0.1
n_estimators	300
max_depth	3
min_samples_leaf	5
min_samples_split	5
subsample	0.8
loss	deviance

### 3.3 Ablation experiment

#### 3.3.1 Ablation experiment of the CAEM

This study conducts ablation experiments to determine the contribution of the CAEM module. In the field of dimensionality reduction, deep learning offers many outstanding methods, including representative ones such as Stacked Autoencoders (SAE), Variational Autoencoders (VAE), and the Convolutional Autoencoder (CAE). These dimensionality reduction methods are combined with Convolutional Block Attention Module (CBAM) in various configurations for ablation experiments. The performance is ultimately evaluated based on accuracy. The experimental results are shown in Table 5.

**TABLE 5:** The results of the CAEM ablation experiments.

	SAE		SAE + CBAM		VAE	
BRCA	Accuracy	82.2	Accuracy	85.7	Accuracy	80.3
	Precision	48.1	Precision	54.2	Precision	46.4
	Recall	50.0	Recall	58.3	Recall	50.0
	F1 score	49.0	F1 score	56.2	F1 score	48.1
GBM	Accuracy	86.0	Accuracy	88.4	Accuracy	85.1
	Precision	58.7	Precision	71.4	Precision	52.1
	Recall	60.4	Recall	62.5	Recall	60.4
	F1 score	59.5	F1 score	66.7	F1 score	55.9
	VAE + CBAM		CAE		CAE + CBAM	
BRCA	Accuracy	83.5	Accuracy	83.4	Accuracy	**95.0**
	Precision	58.9	Precision	52.6	Precision	**97.6**
	Recall	50.0	Recall	45.8	Recall	**88.9**
	F1 score	54.1	F1 score	49.0	F1 score	**93.0**
GBM	Accuracy	87.6	Accuracy	86.8	Accuracy	**91.2**
	Precision	71.2	Precision	62.8	Precision	**91.0**
	Recall	60.4	Recall	60.4	Recall	**90.6**
	F1 score	65.4	F1 score	61.6	F1 score	**90.8**

Bold values represent the best values.

From Table 5, it can be observed that both in terms of individual accuracy and overall F1 score, CBAM demonstrates significantly superior performance across both datasets. Particularly noteworthy is its improvement in CAE, where it achieves nearly a 10% increase in performance on the BRCA dataset. On the BRCA dataset, the accuracy of the other five models is below 90%, while CAEM achieves an accuracy of up to 95%. In contrast, the proposed CAEM model exhibits superior performance. On the GBM dataset, except for the high accuracy achieved by the combination of SAE and CBAM, the accuracy of the other models is significantly lower than that of the CBAM model. Although the combination of SAE and CBAM achieves an accuracy of 88.4%, our proposed CAEM model demonstrates significantly better performance, achieving an accuracy of 91.2%. Furthermore, the CAEM module exhibits good robustness on both datasets. Therefore, the CBAM module can enhance the classification performance of the model to some extent. These experiments above serve as evidence for the effectiveness of the CBAM mechanism.

#### 3.3.2 Ablation study of the attention mechanism

The channel attention module aims to enhance specific channel features by assigning weights to each channel. By applying channel attention first, we can effectively enhance the channel features that are more important for the task. On this basis, the spatial attention module can further focus on important regions in the spatial dimension, thereby further improving the quality of feature representation. We have experimentally demonstrated the effectiveness of this order. Using multi-omics data from the BRCA dataset as an example and taking accuracy, precision, recall, and F1 score as evaluation criteria, the experimental results are shown in Table 6.

**TABLE 6:** Attention mechanism experiment.

	Accuracy	Precision	Recall	F1 score
Channel Attention First	**95.0**	**97.6**	**88.9**	**93.0**
Spatial Attention First	85.0	78.6	75.2	76.9
Using both simultaneously	89.0	95.6	77.8	85.7

The experimental results indicate that the order of applying channel attention first followed by spatial attention performs better on various evaluation metrics. This validates the effectiveness of our design choice.

Bold values represent the best values.

### 3.4 Comparison experiment of dimensionality reduction methods

In CAEM-GBDT, the hierarchical structure CAEM serves as the input for the GBDT classification model. This paper compares it with three dimensionality reduction methods: SAE, NMF, and PCA. The evaluation is performed using these two datasets as input to assess the performance of CAEM in learning features. Classification accuracy is used as the criterion for judging the effectiveness of the dimensionality reduction methods.

From Table 7, it can be seen that in terms of runtime, the training time for one epoch is similar among the three methods except for NMF. Although CAEM-GBDT requires a larger amount of memory, it does not consume more runtime. Moreover, it achieves significantly better results compared to other dimensionality reduction methods. The accuracy of the proposed CAEM dimensionality reduction method is significantly higher than the other three methods. In BRCA dataset, CAEM’s accuracy is notably higher than other dimensionality reduction methods, reaching 95.0%. Similarly, CAEM performs exceptionally well on the GBM dataset. Therefore, this demonstrates the superiority of CAEM for dimensionality reduction. In terms of learning features and achieving dimensionality reduction, CAEM demonstrates the best performance, followed by SAE, NMF, and PCA. The deep learning CAEM dimensionality reduction method can effectively integrate multiple omics data, which will be beneficial for the classification of cancer subtypes.

**TABLE 7:** Details of ablation experiment results.

	SAE		NMF		PCA		CAEM	
BRCA	Accuracy	82.2	Accuracy	83.5	Accuracy	83.4	Accuracy	**95.0**
	Precision	48.1	Precision	62.1	Precision	60.9	Precision	**97.6**
	Recall	50.0	Recall	60.0	Recall	55.0	Recall	**88.9**
	F1 score	49.0	F1 score	61.0	F1 score	57.8	F1 score	**93.0**
	Time	**1.63s**	Time	620.95s	Time	2.29s	Time	1.96s
	Memory	866.5M	Memory	479.8M	Memory	**180.3M**	Memory	0.98G
GBM	Accuracy	86.0	Accuracy	87.6	Accuracy	86.8	Accuracy	**91.2**
	Precision	58.7	Precision	70.1	Precision	69.2	Precision	**91.0**
	Recall	60.4	Recall	68.8	Recall	65.3	Recall	**90.6**
	F1 score	59.5	F1 score	69.4	F1 score	67.2	F1 score	**90.8**
	Time	**1.55s**	Time	193.7s	Time	2.22s	Time	2.32s
	Memory	568.3M	Memory	439.4M	Memory	**396.2M**	Memory	439.5M

Bold values represent the best values.

### 3.5 Comparison experiment with other models

To demonstrate the performance of GBDT, this study employs four different classification models as the final classifiers. The low-dimensional data representation processed by the CAEM module is used as input for each of the four classifiers, with accuracy serving as the evaluation metric.

Furthermore, CAEM-GBDT compared its classification accuracy with other methods, namely, the HI-DFN forest method, which is compatible with multi-omics data as model input, and the DCGN (Shen et al., 2022) method, which is not compatible with multi-omics data as input. In this paper, We evaluated the performance of these two methods as well as four classifiers using the same dataset, and the experimental results are shown in Tables 8, 9.

**TABLE 8:** Comparison of classifier and other model experimental Results (BRCA).

Datasets	BRCA							
	Gene Expression		DNA methylation		miRNA Expression		multi-omics	
CAEM + KNN	Accuracy	70.6	Accuracy	70.6	Accuracy	76.5	Accuracy	82.4
	Precision	43.3	Precision	37.5	Precision	46.2	Precision	48.0
	Recall	35.4	Recall	45.8	Recall	47.9	Recall	50.0
	F1 score	39.0	F1 score	41.2	F1 score	47.0	F1 score	49.0
CAEM + SVM	Accuracy	76.5	Accuracy	70.6	Accuracy	76.5	Accuracy	81.6
	Precision	45.8	Precision	45.0	Precision	37.1	Precision	46.0
	Recall	45.8	Recall	37.5	Recall	43.8	Recall	50.0
	F1 score	45.8	F1 score	40.9	F1 score	40.2	F1 score	47.9
CAEM + RF	Accuracy	76.5	Accuracy	64.7	Accuracy	82.4	Accuracy	82.4
	Precision	37.8	Precision	43.8	Precision	47.7	Precision	47.9
	Recall	47.9	Recall	37.5	Recall	45.8	Recall	47.9
	F1 score	42.3	F1 score	40.4	F1 score	46.7	F1 score	47.9
CAEM +GC forest	Accuracy	82.4	Accuracy	76.5	Accuracy	82.4	Accuracy	84.2
	Precision	46.4	Precision	48.1	Precision	44.6	Precision	47.7
	Recall	50.0	Recall	37.5	Recall	47.9	Recall	58.3
	F1 score	48.1	F1 score	42.1	F1 score	46.2	F1 score	52.5
HI-DFN forest	Accuracy	80.8	Accuracy	73.1	Accuracy	76.9	Accuracy	84.6
	\		\		\		\	
	\		\		\		\	
	\		\		\		\	
DCGN	Accuracy	86.8	\		\		\	
	Precision	83.3	\		\		\	
	Recall	66.7	\		\		\	
	F1 score	74.1	\		\		\	
GBDT	Accuracy	**94.1**	Accuracy	**82.4**	Accuracy	**88.2**	Accuracy	**95.0**
	Precision	**98.1**	Precision	**46.4**	Precision	**71.4**	Precision	**97.6**
	Recall	**87.5**	Recall	**50.0**	Recall	**62.5**	Recall	**88.9**
	F1 score	**92.5**	F1 score	**48.1**	F1 score	**66.7**	F1 score	**93.0**

Bold values represent the best values.

**TABLE 9:** Comparison of classifier and other model experimental Results (GBM).

Datasets	GBM							
	Gene Expression		DNA methylation		miRNA Expression		multi-omics	
CAEM + KNN	Accuracy	67.7	Accuracy	47.0	Accuracy	54.3	Accuracy	70.4
	Precision	72.8	Precision	42.0	Precision	53.6	Precision	75.0
	Recall	66.7	Recall	42.7	Recall	53.1	Recall	67.7
	F1 score	69.6	F1 score	42.3	F1 score	53.3	F1 score	71.2
CAEM + SVM	Accuracy	83.5	Accuracy	50.0	Accuracy	52.9	Accuracy	84.8
	Precision	82.3	Precision	51.9	Precision	51.3	Precision	82.9
	Recall	80.2	Recall	50.0	Recall	52.1	Recall	83.3
	F1 score	81.2	F1 score	50.9	F1 score	51.7	F1 score	83.1
CAEM + RF	Accuracy	83.0	Accuracy	57.2	Accuracy	48.9	Accuracy	84.5
	Precision	79.6	Precision	52.7	Precision	46.3	Precision	83.1
	Recall	77.1	Recall	57.3	Recall	46.9	Recall	82.3
	F1 score	78.3	F1 score	54.9	F1 score	46.6	F1 score	82.7
CAEM +GC forest	Accuracy	86.0	Accuracy	59.2	Accuracy	56.7	Accuracy	87.6
	Precision	85.8	Precision	55.0	Precision	54.7	Precision	85.0
	Recall	86.5	Recall	56.3	Recall	58.3	Recall	85.4
	F1 score	86.1	F1 score	55.6	F1 score	56.4	F1 score	85.2
HI-DFN forest	Accuracy	86.5	Accuracy	59.6	Accuracy	53.9	Accuracy	88.5
	\		\		\		\	
	\		\		\		\	
	\		\		\		\	
DCGN	Accuracy	85.9	\		\		\	
	Precision	82.5	\		\		\	
	Recall	90.0	\		\		\	
	F1 score	86.1	\		\		\	
GBDT	Accuracy	**88.2**	Accuracy	**67.7**	Accuracy	**61.8**	Accuracy	**91.2**
	Precision	**87.6**	Precision	**66.6**	Precision	**54.1**	Precision	**91.0**
	Recall	**88.5**	Recall	**66.7**	Recall	**57.3**	Recall	**90.6**
	F1 score	**88.0**	F1 score	**66.6**	F1 score	**55.7**	F1 score	**90.8**

Bold values represent the best values.

As shown in Tables 8, 9, it is evident that GBDT outperforms other classifiers. In the BRCA dataset shown in Table 6, the accuracy of GBDT using multi-omics data is 95.0%, whereas KNN is 82.4%, SVM is 81.6%, RF is 82.4%, and GC forest is 84.2%. Similarly, in Table 7, the best-performing model in the GBM dataset is also GBDT, particularly achieving an accuracy of 91.2% on multi-omics data, significantly surpassing other classifiers. Therefore, GBDT is selected as the final classifier module in the model.

First, regarding single-omics data, CAEM-GBDT achieves high accuracy across all three omics data types in both datasets. Particularly in the BRCA dataset, whether using single-omics or multi-omics data, CAEM-GBDT improves accuracy by nearly 10% compared to other methods. Meanwhile, the F1 score is also far higher than other methods, demonstrating CAEM-GBDT’s robust performance. In the GBM dataset, CAEM-GBDT also shows improvements over existing methods, especially in DNA methylation data, where the accuracy improvement reaches 8.1%.

In comparison with the HI-DFN forest model, the results of the model were provided by their paper, so evaluation indicators such as F1 scores were not obtained. CAEM-GBDT exhibited high accuracy in all aspects, especially on the multi-omics data of the BRCA dataset, where the accuracy reaches 95%, nearly 10% higher than the HI-DFN forest. In comparison with the DCGN method, DCGN is a cancer subtype identification method exclusively designed for gene expression data. This method exhibits good accuracy and stability in gene expression datasets but cannot handle multi-omics data or other omics data as input. Whether using single-omics data or multi-omics fused data, CAEM-GBDT demonstrates superior performance and better robustness.

From the overall trend in the tables, it can be observed that when the model uses single-omics data as input, the accuracy and F1 score are not as high as those with multi-omics data. Although the accuracy on gene expression data in the BRCA dataset reaches 94.1%, it still improves to 95% when combined with the other two omics data types. This phenomenon is particularly evident in the GBM dataset, where the accuracies of gene expression data, DNA methylation data, and miRNA expression data are 88.2%, 67.7%, and 61.8% respectively. Each omics data type alone has low accuracy, but when combined, the accuracy reaches 91.2%. This demonstrates the impact of multi-omics data on cancer subtype identification results. This trend is not only observed in CAEM-GBDT but also in other models. Therefore, it can be concluded that multi-omics data, compared to single-omics data, enables the model to learn complementary information between omics data, achieving higher accuracy and F1 scores for cancer subtype identification. Moreover, the results are more convincing as they integrate various omics data, benefiting cancer subtype identification.

## 4 Discussion

With the advancement of sequencing technologies, integrating multi-omics data for cancer subtype identification is a major challenge faced by researchers. This paper proposes a cancer subtype classification method, named-GBDT, based on Convolutional Autoencoder and Convolutional Attention. It aims to accurately extract crucial information contained in complex multi-omics data. The Convolutional Attention Mechanism is employed to further enhance features in the high-level representation, enabling the classifier to more effectively identify important hidden information in the data and perform cancer subtype classification. Compared to other deep learning methods published at the current stage, CAEM-GBDT has the following characteristics:1. CAEM-GBDT designs different encoder networks for different data settings. It can handle individual omics data as well as fuse multiple omics data as input to the model, achieving high accuracy in both scenarios.2. CAEM-GBDT attempts to learn relationships between multiple omics data, fully leveraging multi-omics information among samples. This enhances the model’s robustness, making the identification results more convincing.

This paper conducted encoder network ablation experiments, dimensionality reduction method comparison experiments, classifier comparison experiments, and comparative experiments with the HI-DFN forest model, demonstrating the powerful capabilities of CAEM-GBDT. Experimental results indicate that the fusion of multi-omics data is effective and necessary. Multi-omics data describe the data from different perspectives, providing the model with more stable performance and enhanced robustness.

Although CAEM-GBDT has demonstrated promising results on the BRCA and GBM datasets, there are still some limitations. CAEM-GBDT is tailored specifically for supervised learning in classification. In the research on unsupervised learning and clustering methods, CAEM-GBDT’s performance is not as satisfactory. Therefore, addressing this limitation will be a key focus of our next research steps.

## 5 Conclusion

This paper proposes a cancer subtype classification method, CAEM-GBDT, based on Convolutional Autoencoder and Convolutional Attention Mechanism. The integration of multiple omics data improves the accuracy of cancer subtype classification. Addressing the challenges posed by high-dimensional and sparse biological datasets, this approach combines Convolutional Autoencoder (CAE) and Convolutional Block Attention Module (CBAM) to obtain effective data representations from cancer data. The results on two TCGA datasets demonstrate that CAEM-GBDT outperforms other methods, showing its superior performance.

## Data Availability

The original contributions presented in the study are included in the article/Supplementary Material, further inquiries can be directed to the corresponding author.
